# Unveiling the Effects of Natural Disasters and Nuclear Energy on the Secondary Sex Ratio: A Comprehensive Review

**DOI:** 10.3390/life15071127

**Published:** 2025-07-17

**Authors:** Iasonas Dermitzakis, Paschalis Theotokis, Efthymia Delilampou, Evangelos Axarloglou, Sofia Gargani, Dimosthenis Miliaras, Maria Eleni Manthou, Soultana Meditskou

**Affiliations:** Department of Histology-Embryology, School of Medicine, Aristotle University of Thessaloniki, 54124 Thessaloniki, Greece; iasonasd@auth.gr (I.D.); ptheotokis@auth.gr (P.T.); edelilamp@auth.gr (E.D.); evanaxar@auth.gr (E.A.); sgargan@bio.auth.gr (S.G.); miliaras@auth.gr (D.M.); mmanthou@auth.gr (M.E.M.)

**Keywords:** secondary sex ratio, natural disaster, nuclear energy, earthquakes, reproductive health, population dynamics, public health

## Abstract

The secondary sex ratio (SSR), defined as the ratio of male to female births in a population, has long been a subject of scientific inquiry due to its potential as a health indicator. The interplay between catastrophic events and the delicate balance of male and female births presents a nuanced and compelling study area. Natural disasters, such as earthquakes, hurricanes, floods, and volcanic eruptions, have been known to disrupt ecosystems and human populations, leading to both short-term and long-term consequences. Studies have suggested a potential influence of these disasters on the SSR, with varying degrees of impact observed across different regions and disaster types. Similarly, nuclear accidents, such as the infamous Chernobyl disaster, have sparked interest in their potential effects on human health and development. The release of radioactive materials into the environment can have far-reaching consequences, including impacts on reproductive outcomes. Through a rigorous examination of the existing literature, the present review aims to synthesize current knowledge on the impacts of natural disasters and nuclear accidents on the SSR and unravel the mechanisms that explain SSR fluctuations. By shedding light on the diverse influences shaping the SSR, this narrative review contributes to a deeper appreciation of the intricate interplay between environmental, biological, and societal factors that determines the SSR, calling for targeted strategies to mitigate potential adverse effects on sex ratios in the aftermath of such events.

## 1. Introduction

The secondary sex ratio (SSR), a fundamental demographic indicator denoting the ratio of male to female live births within a population, serves as a pivotal point of interest in the field of reproductive biology and public health [1]. Globally, the average SSR stands at around 1.05, revealing a slight predominance of male newborns compared to females. It is crucial to acknowledge that notable disparities in the SSR can manifest across various nations and geographic areas [2]. Nevertheless, the balance between male and female offspring is influenced by a multitude of factors, both biological and environmental, making it a subject of profound intrigue amongst scientists and policymakers worldwide [3]. More precisely, factors such as parental age [4], maternal weight [5], birth order [6], seasonal variations [7], socioeconomic conditions [8], terrorism [9], wars [10], anxiety disorders [11], exposure to chemicals [12], parental occupation [13], infections [14], natural disasters [15], and nuclear accidents have all been recognized as potential influences contributing to trends in the SSR [16].

Fluctuations in the SSR can be influenced by factors that come into play either at conception or during gestation [17]. Over the years, there has been ongoing debate surrounding the theory that governs gender determination at conception [18,19]. Typically, X- and Y-bearing sperm have an equal chance of fertilizing an egg, with the presence of the Y chromosome determining male fetal development [20,21]. However, certain factors affecting the reproductive systems of either the father or mother can lead to an imbalance in the sex ratio through distinct biological mechanisms (e.g., sperm motility) [22]. It is believed that higher levels of sex-specific parental hormones like estrogen and testosterone are linked to the birth of male offspring, whereas increased levels of progesterone and gonadotrophins are associated with the birth of female offspring [23]. Despite these associations, the exact mechanism of hormonal influence affecting the sex ratio at conception remains unclear. Conducting direct experimental testing in humans to validate this hypothesis is ethically challenging [24].

Following conception, the viability and growth of embryos in the uterus can also impact the SSR [24]. Specific critical periods have been identified as a result of the profound impact of stress on cellular and tissue differentiation [25,26,27,28,29]. Maternal stress is a key factor in in utero selection, with increased secretion of maternal adrenal cortex androgens, specifically from the zona reticularis, heightening the risk of miscarriage [30,31]. Given that miscarriages in such circumstances tend to be biased towards male fetuses, an imperative investigation into the underlying causes is warranted [32]. One possible rationale lies in the sex-specific ramifications of testosterone. While maternal testosterone levels did not exhibit disparities based on fetal sex, notable effects on the development of male offspring were apparent, which contrasted with the absence of statistically significant correlations in females [33]. Concerning male-biased miscarriages, it is essential to consider evolutionary theories like the Trivers–Willard hypothesis, maternal immunoreactive responses to male fetuses, the prolonged vulnerability of male embryos, and the heightened susceptibility of the male placenta to stress [34]. The Trivers–Willard hypothesis postulates that the condition of parental health tends to bias the SSR towards sons in favorable circumstances and towards daughters in adverse conditions under the influence of natural selection [35,36].

Among the factors impacting the SSR, natural disasters and nuclear accidents have gained considerable attention due to their far-reaching consequences in human populations. The aftermath of events like earthquakes, hurricanes, floods, and volcanic eruptions often leaves a profound imprint on communities, affecting not only the physical and psychological well-being of individuals but also the demographic composition of populations [37,38]. Along the same line, nuclear accidents such as the Chernobyl disaster in 1986 and the Fukushima nuclear disaster in 2011 have raised concerns about their potential impacts on human health and reproductive outcomes [39]. Exposure to ionizing radiation, a common consequence of nuclear accidents, can pose risks to a developing fetus and influence the SSR [40]. Understanding changes in the SSR following catastrophic events is important because such shifts may reflect underlying biological mechanisms shaped by natural selection to optimize population fitness when under stress. These variations can serve as sensitive indicators of population-level responses to environmental adversity, with implications for public health surveillance, risk assessment, and long-term demographic planning.

The present narrative review aims to summarize the existing literature on SSR trends in the context of both natural and nuclear disasters. By encompassing a broad spectrum of disaster types—including earthquakes, cyclones, floods, volcanic eruptions, and nuclear incidents—across diverse geographic regions, this review endeavors to elucidate patterns of SSR fluctuations under varying environmental stress conditions. Through an analysis, our study seeks to offer valuable insights for researchers, policymakers, and practitioners in the fields of public health, disaster management, and reproductive biology.

## 2. Review Methodology

This comprehensive narrative review was conducted to explore the potential impacts of natural disasters and nuclear energy on the SSR. Although the present work did not adhere to the rigorous protocols of a systematic review, a structured approach was employed to ensure the inclusion of relevant and high-quality sources. An extensive literature search was performed using academic databases, including PubMed, Scopus, and Web of Science, with the aim of identifying studies examining associations between natural disasters, nuclear events, and SSR outcomes. The search terms included combinations of keywords such as “secondary sex ratio”, “sex ratio at birth”, “natural disasters”, “earthquakes”, “cyclones”, “volcanic eruptions”, “floods”, “nuclear energy”, “nuclear accidents,” and “radiation exposure.” Eligible articles included peer-reviewed original research written in English with full-text availability. The reference lists of the included studies were manually screened to identify additional sources. Studies were selected by assessing titles and abstracts, followed by a full-text review to confirm relevance. No formal quality appraisal tool was employed, but methodological limitations, confounding factors, and potential biases were noted and are addressed in the discussion where applicable. Data were extracted for key parameters such as the type and date of the disaster or nuclear event, the population studied, the gestational timing of the exposure, the direction and timing of the SSR change, and proposed biological mechanisms. The findings were synthesized narratively to highlight patterns, divergences, and gaps in the existing literature, with an emphasis on potential pathophysiological explanations and public health implications.

## 3. Natural Disasters

Natural disasters, in general, are defined as “large-scale adverse events resulting from natural processes of the earth, often associated with death, trauma, and destruction of property” [35]. These events have a profound impact and, at times, a long-lasting influence on the mental health of the affected population [36].

### 3.1. Earthquakes

Earthquakes, defined by the World Health Organization as “violent and abrupt shaking of the ground, caused by movement between tectonic plates along a fault line in the earth’s crust”, are significant and life-threatening incidents [41]. Their consequences for public health, including casualties, injuries, psychological distress, and the destruction of healthcare facilities, are often immeasurable [42,43]. Their psychological effects are profound and long-lasting, stemming from the loss of relatives, livelihoods, and properties, and they can ultimately contribute to a higher prevalence of post-traumatic stress disorder [44]. Such an external stress factor can consequently impact the SSR of offspring born in the subsequent months [31,36,45].

#### 3.1.1. The 1999 Gölcük Earthquake

Several studies have examined the impacts of major earthquakes on the SSR. Initially, Doğer et al. researched the influence of the Gölcük earthquake in Eastern Marmara, Turkey, which occurred on 17 August 1999, with a magnitude of 4.2 R [46]. This incident resulted in numerous deaths and extensive destruction of property [47]. Monthly records of live births were collected from January 1997 to December 2002, and the SSR was calculated for each month. As male fetuses are more susceptible to miscarriages due to elevated maternal stress levels, a decrease in the SSR within a period of fewer than nine months would indicate a postconceptional effect of the earthquake [48,49]. The results showed significant reductions in the SSR four and eight months after the earthquake, specifically in December 1999 and April 2000. The variation noted in December could be attributed to the mandatory 30-day notice period required for birth registration in Turkey and the prevalent practice of registering December-born babies with an age one year younger in official records. The decline observed in male infants in the affected region in April 2000, as opposed to male infants in April 2001 and April 2002, was also statistically significant. These data validate that such events, which cause intense and widespread psychological distress, can lead to spontaneous abortions of male fetuses [50].

#### 3.1.2. The 2001 Gujarat Earthquake

Furthermore, on 26 January 2001, a massive earthquake measuring 7.7 on the Richter scale devastated Gujarat, India, resulting in numerous casualties and extensive property damage [51,52]. To assess the earthquake’s impact, data from District-Level Household Surveys was retrieved, and births spanning from 1996 to 2006 were included. The study revealed a significant decrease in the SSR of 4.1% for the 21 affected districts compared to the pre-earthquake value [53]. Nevertheless, this impact was significant at the 10% level. In an extended analysis encompassing longer periods, specifically 1994–2000 and 2002–2008, the earthquake resulted in a reduction of 1.9 percentage points in the rate of male births among rural women, which was statistically significant at the 1% level. There was no discernible impact on the SSR within the overall sample. No fluctuations in the male birth rate were detected in shorter study intervals (1998–2000 and 2002–2004). However, when the researchers included the year 2001 in their dataset, considering the periods of 1996–2000 as pre-earthquake and 2001–2005 as post-earthquake, this catastrophic event led to a decrease of 2.3 percentage points in the rural male birth rate, which was also statistically significant at the 1% level. According to all the findings mentioned above, the SSR decline can be attributed to the Trivers–Willard hypothesis.

#### 3.1.3. The 2003 Bam Earthquake

Another devastating earthquake measuring 6.6 on the Richter scale, resulting in the tragic loss of over 20,000 lives and extensive destruction of property and civic buildings, struck Bam City, Kerman Province, Iran, on 26 December 2003 [54]. In light of this shocking event, Saadat conducted a study to examine the potential impact on the SSR 6–12 months following the Bam earthquake [55]. To gather data, the study utilized monthly birth records from the National Organization of Civil Registry for the subsequent 12 months, as well as a control group of records spanning 33 months prior to the earthquake. The findings revealed a notable decline in the SSR that reached statistical significance in Bam City, specifically in November 2004, approximately 11 months after the earthquake. However, the SSR did not demonstrate a significant decrease in the broader records from Kerman Province during the same period. Consequently, the author proposed that the socioeconomic crisis resulting from the complete devastation of the city’s central area may have led to extended psychological distress, potentially impacting the periconceptional period [56,57].

#### 3.1.4. Seismic Sequence on Zakynthos Island

From 3 April to 8 May 2006, Zakynthos Island in Greece experienced prolonged earthquakes. These earthquakes had mediocre magnitudes ranging from 5.1 to 5.6 on the Richter scale; fortunately, no casualties were reported [58]. In their research, Tourikis and Beratis gathered data on monthly birth records from the Statistical Bureau of Greece for the months following the earthquake sequence. Interestingly, they found that the decline in the SSR was generally delayed compared to previous estimates. Within nine months following the seismic sequence, a decline in the SSR was observed in February 2007. The most profound decrease occurred two months later, in April. These findings suggest that the decline in the SSR occurred later than expected, indicating a potential influence of the earthquakes during the periconceptional stage. According to previous studies mentioned by the researchers, three potential etiological pathways could be responsible for this effect: a reduction in coital frequency, hormonal imbalances during conception, and reduced motility of Y spermatozoa. Furthermore, the researchers note that the previous history of a major seismic event in this region in 1953 may have contributed to psychological distress among the population due to fear of similar casualties [59]. This psychological distress could also have played a role in the profound decline in the SSR.

#### 3.1.5. The 2009 L’Aquila Earthquake

The impact of earthquakes during the periconceptional period was once again validated in the case of L’Aquila, Italy, on 6 April 2009 [60]. The magnitude of this seismic event was 6.3 R. Researchers obtained birth records from the Clinical Obstetrics and Gynecology Department of the San Salvatore Hospital. Specifically, they retrieved data from 1 January to 30 June 2010 (post-earthquake) and from 1 January to 30 June 2008 (pre-earthquake). The findings indicated that the L’Aquila earthquake had a negative effect on the SSR nine months later, precisely in January 2010. However, this decline was not statistically significant. Significant statistical variances in the SSR were only observed when comparing the first trimester of 2010 to the second trimester. The researchers attribute these findings to both unsuccessful conception with Y spermatozoa (resulting in a lower number of male births) and the increased vulnerability of male fetuses when maternal stress levels are elevated [49].

#### 3.1.6. The 2011 Tohoku Earthquake

In Japan, seismic events are relatively common and can reach significant magnitudes [61]. One of the most devastating natural disasters in recent history was the Tohoku earthquake (also known as the Great East Japan earthquake) on 11 March 2011, with a tremendous magnitude of 9.1 on the Richter scale. This event caused a destructive tsunami and a nuclear accident at the Fukushima nuclear facility [62]. The psychological distress experienced by those affected by the disaster had a lasting impact [63].

**Figure 1:**
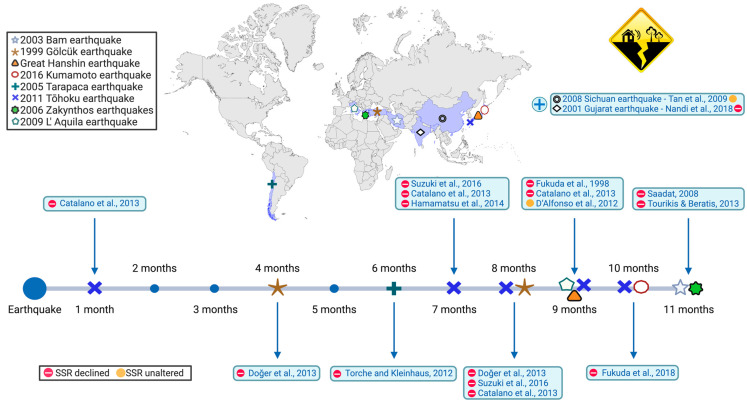
The effects of earthquakes on the secondary sex ratio (SSR). Each examined earthquake is denoted by a specific symbol displayed in the uppermost box. The time required to observe a shift in the SSR following each earthquake is depicted. A red bullet indicates a statistically notable SSR decline, while a yellow bullet indicates no significant alteration in the SSR. The studies related to the 2008 Sichuan earthquake and 2001 Gujarat earthquake did not refer to specific times regarding alterations in the SSR; thus, they are depicted separately. The figure was created on BioRender.com.

In a study following the Tohoku earthquake, monthly birth data from 1997 to 2012 were collected for all prefectures in Japan, including regions affected and not affected by earthquakes [64]. Upon analyzing the data, the researchers discovered a significant decline in the SSR across all regions of Japan seven months after the earthquake, specifically in October 2011 (Figure 1). Interestingly, the SSR in the non-affected regions dropped to an even lower level compared to the regions directly impacted by the earthquake. The study also highlighted that the entire Japanese population experienced high stress levels due to ongoing fears regarding health consequences resulting from the nuclear accident. Furthermore, the authors suggest that the economic crisis resulting from the shutdown of all nuclear facilities in the country added to the existing uncertainty about the future. The decrease in the SSR observed seven months later in all prefectures can be attributed to the in utero selection mechanism. The smaller decline in the SSR observed in the affected regions may be a result of increased reproductive activity among couples aiming to replace lost descendants.

In a similar study, data on monthly births were collected from March 2010 to March 2012 [65]. The study found a decline in the SSR among women living in severely affected prefectures during early pregnancy (4–11 weeks of gestation) in 2011. Interestingly, there was also a statistically significant decline in the SSR for similar women in unaffected regions. However, there was no significant difference in the SSR for women exposed to the earthquake during late gestation (28–36 gestational weeks). As the impact of this catastrophic event occurred in gestational weeks 4–11, the decline in the SSR was noticeable after approximately seven to eight months. These findings can be interpreted in accordance with the selection in utero theory [45]. Fukuda et al. conducted a study on the same topic [66]. They found that the SSR in Miyagi Prefecture, the region most affected by the Tohoku earthquake, was significantly lower. Declines were observed in December 2011 and January 2012, nine and ten months after the natural disaster. The SSR in January 2012 was statistically significantly lower than in the previous January. The authors suggest that the reduction in the conception of males could be attributed to reduced sperm motility, similar to what was observed following the Kobe earthquake [22].

Additionally, Catalano et al. conducted a study on the impact of the Tohoku earthquake on the SSR [67]. They found that the number of male births in the three most affected prefectures was significantly lower in April, October, November, and December 2011. The authors propose that both decreased male conception and adverse in utero conditions contributed to the decline in the SSR. The decrease in male births in October and November suggests a higher loss rate of frail male fetuses, while the decreased SSR in December 2011 indicates that acute stress following a natural disaster can also affect the conditions for conception, resulting in fewer male conceptions. The immediate decline in male births in April 2011, one month after the Tohoku earthquake, suggests late occurrences of spontaneous abortions of male fetuses due to adverse intrauterine conditions. Only the prefectures most distant from the epicenter and least affected by the earthquake did not show significant declines in the SSR within the first months after the earthquake. The SSR decreased in October. Other regions of Japan, excluding the most affected and distant prefectures, presented notable SSR reductions in March, June, and October.

#### 3.1.7. Great Hanshin Earthquake

The Great Hanshin earthquake, also known as the Kobe earthquake, was a significant natural disaster that occurred on 17 January 1995, with a magnitude of 7.2 on the Richter scale, resulting in over 5500 fatalities [68]. Its impact on the population was immediate and long-lasting, causing high stress levels and affecting the overall socioeconomic conditions [69,70]. With this in mind, a retrospective study was conducted three years after the earthquake to explore potential effects on the SSR [71]. This study collected data on recorded monthly births from Hyogo Prefecture, explicitly focusing on data from 6 to 12 months after the earthquake (June 1995 to January 1996). This data was then compared to the corresponding period two years prior to the earthquake (June 1993 to January 1994), which served as a control group for comparison purposes. Remarkably, a statistically significant decrease in the SSR was observed in October 1995, precisely nine months after the earthquake. Importantly, there was no notable difference in the number of spontaneous abortions compared to previous years. Based on these findings, the authors suggested that the earthquake affected the periconceptional period. In addition to this retrospective study, the same research team conducted an experimental study in 1996, focusing on males from the Kobe population [22]. This study aimed to investigate semen quality following the earthquake. The results revealed that out of the 27 men examined, 10 exhibited statistically significantly lower sperm motility within five months after the natural disaster. This finding led the researchers to hypothesize that if this reduced motility predominantly affected Y spermatozoa, it could explain the decline in the SSR.

#### 3.1.8. The 2016 Kumamoto Earthquake

The Kumamoto earthquake, which took place on 16 April 2016, had a magnitude of 7 on the Richter scale and tragically led to around 300 fatalities [72]. A study examining the impact of this natural disaster on the SSR revealed a significant decline in the SSR around ten months after the Kumamoto earthquake, specifically in February 2017 [66]. This decline occurred more than nine months following the incident, implying that major stress events can affect the conditions of conception. Consequently, the authors suggest a potential negative effect on Y spermatozoa after paternal exposure to such stressors, resulting in a lower rate of successful fertilization of the ovum. Lastly, Fukuda et al. proposed a new pathophysiological explanation for the effects of several major earthquakes on the SSR [15]. The authors hypothesized that elevated stress during natural disasters can disrupt maternal immune tolerance during pregnancy, selectively affecting male fetuses. H-Y antigens, a group of minor histocompatibility antigens that are located on the Y chromosome and have high immunogenic activity, serve as the basis for this theory [73]. Several findings support this hypothesis, showing a correlation between increased H-Y antibodies and a decreased SSR [74,75].

#### 3.1.9. The 2005 Tarapaca Earthquake

Fluctuations in the SSR can be influenced by sex-selective preterm births, as observed in a registry following the Tarapaca earthquake in Chile, which was characterized by a magnitude of 7.9 on the Richter scale. Researchers Torche and Kleinhaus examined the impacts of earthquakes on women in the postconceptional stage [76]. They found that exposure during the third month of gestation led to a decline in the recorded SSR of the affected regions, although this reduction was not statistically significant. A notable downshift in the SSR was identified among females exposed to the earthquake in the eighth month of gestation. No changes in the SSR were reported in the unaffected regions. Additionally, the researchers observed that earthquake exposure resulted in a significantly reduced gestational age for female fetuses compared to male embryos. Therefore, when considering the counterfactual SSR values (i.e., the expected value of the SSR if the earthquake did not affect gestational age), women who experienced the earthquake during their third month of gestation exhibited a significant SSR decline of 5.8%. As a consequence of this catastrophic event occurring in the third gestational month, the decline in the SSR was noticeable after five months. The disparity in statistical significance between the recorded and counterfactual SSRs suggests an increased likelihood that preterm female births contributed to the deviation from the expected decline in the SSR.

#### 3.1.10. The 2008 Sichuan Earthquake

On the other hand, the Sichuan earthquake, also known as the Wenchuan earthquake, which took place on 12 May 2008, with an estimated magnitude of 7.9 R, was one of China’s most devastating natural disasters [77]. Such highly stressful events during the perinatal period can have impacts on various birth outcomes, including birth weight and the presence of birth defects [78,79]. In order to study these outcomes in the first year after the Sichuan earthquake, Tan et al. conducted a study until 11 May 2009 [80]. The control group consisted of birth records from the previous year, from 12 May 2007 to 11 May 2008. While significant differences were observed in birth weight, preterm births, and the occurrence of birth defects, there was no statistically significant decrease in the SSR.

To sum up the findings discussed above, they collectively suggest that earthquakes decrease the SSR, primarily due to the influence of psychological distress (Figure 1). This distress can consequently lead to a higher percentage of male spontaneous abortions, as male fetuses are more vulnerable to in utero exposure to maternal stress. Additionally, hormonal imbalances and decreased motility of Y spermatozoa serve as potential mechanisms for the impact of earthquakes on the SSR at the time of conception.

### 3.2. Cyclones and Hurricanes

Cyclones and hurricanes are highly destructive natural disasters that wreak havoc on the affected populations, resulting in substantial surges in both mortality and morbidity rates. These catastrophic events inflict widespread damage and loss of life, leaving a lasting impact on the affected communities [81,82]. The detrimental consequences of cyclones and hurricanes are evident in the increased vulnerability of individuals, compromised healthcare systems, and the long-term health and socioeconomic implications the affected regions face [83].

#### 3.2.1. Cyclones Yasi and Marcia

A study conducted by Parawiya et al. aimed to examine the impact of two major Australian cyclones, Yasi and Marcia, which occurred in February 2011 and February 2015, respectively [84]. The researchers divided the study population into three groups, namely pregnant women exposed to the natural disasters during the early, middle, and late stages of pregnancy. They collected birth records from national databases from 2007 to 2018, focusing on the nine months following these catastrophic events. The study found a statistically significant increase in the SSR for pregnant women exposed to the Yasi cyclone in the first trimester. Thus, this SSR increase was noticeable seven to nine months after the catastrophic event. Additionally, there was an upshift in the proportion of male births for women facing the Marcia cyclone in the middle stage of pregnancy, but this increase was not statistically significant. Furthermore, the study observed an increase in the ratio of male stillbirths for women exposed to the Yasi cyclone during the middle stage of pregnancy and those that experienced the Marcia cyclone during the late stage of pregnancy. However, these increases did not reach statistical significance. The authors attributed these findings to the increase in the in utero female mortality rate after cyclones, particularly in the early stages of gestation. Elevated maternal stress levels might render female fetuses more vulnerable compared to males. This stands in contrast to the commonly referenced notion of a “male disadvantage” in utero. Therefore, further investigation is deemed necessary to delve into the repercussions of heavy rainfall on female fetuses during pregnancy.

#### 3.2.2. Hurricane Katrina

Hurricane Katrina occurred on 25 August 2005 and caused unprecedented destruction in New Orleans, affecting over 1 million people [85,86]. A retrospective study was conducted to test the hypothesis of a reduction in the SSR after the hurricane by collecting data from approximately 0.5 million assisted reproductive technology (ART) cycles [87]. The study also recorded the follicular stimulating hormone (FSH) dosage used for stimulation and the number of retrieved oocytes. Exposure to the natural disaster occurred entirely before conception, as the in vitro fertilization cycles started in September 2005. As a result, there was a decline in the SSR from 51% to 49.4%, but this reduction was not statistically significant. Following the hurricane, higher FSH stimulation dosages were required for success in the ART process, and there was also a higher prevalence of diminished ovarian reserve diagnoses. Similarly, Long et al. demonstrated no significant link between Hurricane Katrina and fluctuations in the SSR after retrieving records from the IBM Health Market Scan dataset and using both seasonal autoregressive integrated moving-average and state-space models [88].

On the contrary, another study revealed that the SSR increased in the four states affected by Hurricane Katrina, specifically Mississippi, Louisiana, Alabama, and Florida [89]. The data used in this analysis were collected from the Centers for Disease Control and Prevention website and included monthly birth records from January 2003 to December 2012. The findings indicate that the increase in the sex ratio was evident approximately 8–10 months after Hurricane Katrina, specifically during the April-to-June period of 2006. Furthermore, the level of severity of the hurricane in each state was a determining factor for the fluctuations in the SSR. For instance, there was no significant difference in the SSR in Florida, where Hurricane Katrina was less intense. At the same time, in the three most affected states, namely Alabama, Louisiana, and Mississippi, the increase in the SSR reached statistical significance. These findings are linked to the theory that heightened background radiation stemming from intense rainfall causes lethal mutations in parental X gametes. Further details regarding the specific impact of radiation on the sex ratio will be provided in the subsequent section regarding nuclear incidents.

Based on the data provided, the impact of cyclones and hurricanes on the SSR is contradictory (see Table 1). Although some studies suggest a trend towards an increase in the SSR, not all reported studies agree. One proposed pathophysiological mechanism that may contribute to this increase is the rise in ambient natural background radiation following heavy rainfall. This increase in radiation may result in greater loss of female fetuses due to lethal mutations in X gametes.

### 3.3. Floods

Floods are highly prevalent natural disasters that have notable implications for human health. They can give rise to various complications, including infections such as leptospirosis, gastroenteritis, and pathological conditions affecting the skin and soft tissues [94,95]. Moreover, the psychological well-being of affected individuals is profoundly affected by such sudden events, often leading to persistent repercussions, like post-traumatic stress disorder, that necessitate clinical intervention [96,97].

#### 3.3.1. The 1965 Brisbane Flood

Research examining the impact of the Brisbane flood of July 1965 was conducted [90]. The study collected birth records from two maternity hospitals in Brisbane, Australia. The findings revealed a significant decrease in the SSR, specifically from 6th to 11th June 1966, approximately 320 days after the 1965 floods. Notably, Lyster highlights a previously established mechanism as a potential explanation for the observed changes in the SSR [98]. The proposal suggests that Y spermatozoa are more vulnerable to environmental conditions such as stress during the post-meiotic stages. It is posited that paternal exposure to such events prior to conception can lead to a higher likelihood of a female-skewed SSR. Furthermore, Lyster suggests that changes in factors such as drinking water pH and nutrient levels might influence the process of spermatogenesis.

#### 3.3.2. The 2010 and 2011 Pakistan Floods

The effects of floods on the SSR in the Pakistani population were also investigated [91]. Two major floods occurred during the summers of 2010 and 2011, affecting 78 out of 121 districts in Pakistan and impacting over 25 million people [99]. Nadir conducted a study to examine the influence of these floods on affected pregnant women. The 2010 flood lasted from July to September, while the 2011 flood occurred from August to September. However, analysis of the birth records from the affected regions revealed that in utero exposure to the natural calamity did not alter the sex ratio at birth.

Thus, floods are common natural disasters with an effect on the SSR that is not well understood. The timing of exposure may play a defining role, as observed in the Brisbane flood study, where exposure to the flood ten to eleven months before birth significantly reduced the SSR. However, in utero exposure to the stressful conditions of floods did not impact the ratio of male offspring.

### 3.4. Volcanic Eruptions

Volcanic eruptions are an exceptional category of natural disasters. Their consequences are far-reaching, affecting the environment, physical health, and mental well-being [100,101]. One notable example of their impact on mental health is the eruption of Eyjafjallajökull volcano in Iceland in April 2010. A psychological survey conducted nine months after the incident revealed significantly increased risk of mental distress, PTSD symptoms, and perceived stress among the local population [102].

#### 3.4.1. The 2010 Eruptions of Eyjafjallajökull

For the same incident, Grech and Borg examined the potential influence of the Eyjafjallajökull volcanic eruptions on the SSR [92]. They anticipated a decline in the SSR, occurring 3–5 months after the eruption, as a result of women’s exposure to a stressful event during pregnancy [103]. After adjusting to account for seasonality, slight decreases in the SSR were noted in September 2010 and November 2010, corresponding to 5 and 7 months following the volcanic eruption. The authors suggest that culling frail male fetuses could be the etiologic mechanism behind these findings [104].

#### 3.4.2. The 1783 and 1784 Eruptions of Laki

Furthermore, Catalano et al. conducted a study examining the impact of the Laki volcanic eruptions in Iceland, which occurred from June 1783 to January 1784, on postconceptional gestations [93]. The researchers considered the unprecedented release of particulate matter and sulfur dioxide gases and the resulting acid rain as significant external stressors that affected the gestational period [76,105]. They conducted a time-series analysis to investigate this, analyzing birth data from 80 cohorts from 1751 to 1830. The study revealed a significant decline of 2.6% in the SSR in 1784 following the volcanic eruptions. Interestingly, the SSR rebounded and showed a compensatory increase of 2.1% 18 years later from 1798 to 1799. The authors also investigated whether the observed decline in the SSR was attributable to an inherited tendency for the population to produce male or female offspring, as suggested by Hamilton’s theory, or if the volcanic eruption influenced it. Their findings suggest that the most plausible explanation for the observed results is the occurrence of spontaneous abortions of the frailest male fetuses, also known as selection in utero [45,104].

In conclusion, volcanic eruptions have psychological consequences in affected populations, in addition to their significant threats to physical health and the environment. The mentioned studies demonstrate a negative impact on the SSR, with the pathophysiological mechanism being induced by elevated maternal stress levels. These stress levels potentially result in selective culling of frail male fetuses.

## 4. Nuclear Energy and Accidents

Nuclear energy is defined as the energy released during nuclear fission or fusion. It has been extensively utilized in modern societies for electricity generation [106]. Numerous nuclear incidents have transpired in nuclear power plants, including the globally renowned accidents in Chernobyl and Fukushima, which have had diverse and not entirely understood impacts on human health [107,108]. Substantial effects on the reproductive system, such as fertility problems and a decrease in the number of offspring, have been reported, thus suggesting that a potential alteration in the SSR would not be irrational [109]. Due to the simultaneous occurrence of the 2011 Tohoku earthquake and the Fukushima nuclear accident, there is a lack of reports that independently detail the impact of this nuclear incident on the SSR. Any study integrating the effects of both the earthquake and the nuclear accident was documented within the Earthquakes Section.

### 4.1. Chernobyl Disaster and Atomic Bomb Tests

Initially, an ecological study examined the potential fluctuations in the SSR resulting from the Chernobyl nuclear incident on 26 April 1986. Researchers collected annual data from eight northern and eastern European countries for the decade spanning from 1982 to 1992 [110]. Combining the measurements from all included countries demonstrated a downward trend during the 1982–1992 period. However, the analysis revealed a notable spike in 1987 after the Chernobyl nuclear accident. Furthermore, elevated thyroid hormone levels were found in the years following the incident [111]. Consequently, the authors propose that the increase in the SSR observed after the Chernobyl accident may be attributed to dysregulation of the hypothalamus–pituitary–thyroid axis in women, which could subsequently influence embryonic and fetal development in utero. Additionally, the authors propose that irradiation may impact paternal X gametes, potentially causing lethal X-chromosome mutations and leading to an increased rate of female loss.

The notion of a selective and more pronounced impact on paternal gametes is supported by a study on the Ukrainian population [112]. Dubrova et al. specifically noted a significant mutagenic effect of ionizing radiation on the male germline, while the mutation rate in maternal sex cells remained unaffected. These observations were derived by analyzing DNA samples from Ukrainian families before and after the Chernobyl disaster, from 1976 to 1996. Thus, this observation could explain the selective in utero abortion of female conceptuses. In line with the findings above, a study investigated the impact of ionizing radiation on the SSR across 39 European countries and the USA [40]. The SSR alterations in both Europe and the USA exhibited a consistent pattern of a uniform decrease from 1950 to 1964, followed by an increase from 1964 to 1975 that could be attributed to the delayed global dispersion of atomic bomb tests prior to the signing of the Partial Test Ban Treaty in 1963. Subsequently, there was a relatively steady decline from 1975 to 1990. In contrast to the USA, Europe experienced a notably significant rise in the SSR of 0.20% in 1987, coinciding with the Chernobyl incident. The authors suggested that if additional data on other factors contributing to the SSR, such as age and social class, were incorporated into the study, the impact of irradiation could be diminished and could become insignificant.

**Figure 2:**
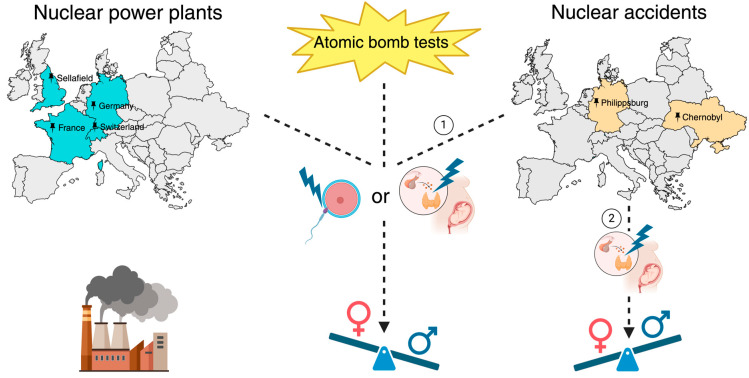
The effects of nuclear energy on the secondary sex ratio (SSR). Operation of nuclear power plants, atomic bomb tests, and nuclear accidents could lead to an increased SSR through lethal mutations in the germline, especially in paternal X gametes, or through thyroid dysregulation in pregnant women. Although most of the studies revealed SSR declines following the Chernobyl disaster (1), one study showed a possible decline in the SSR due to this incident (2). The figure was created on BioRender.com.

In the most recently published data concerning the fluctuations in the SSR resulting from the impact of the Chernobyl incident, a detailed examination was undertaken in Italy [16]. Two notable increases in the SSR were observed among a total of 57.7 million births spanning from 1940 to 2019. Specifically, one increase was observed in the late 1960s following a series of nuclear atomic bomb tests, and the second increase occurred in 1987 after the Chernobyl incident. In both cases, the elevated SSR was associated with higher exposure to Cesium (Cs) radioisotopes, specifically Cs-134 and Cs-137. This study further implicates the heightened vulnerability of paternal X chromosomes as the most likely pathophysiological mechanism. In addition to the potential culling of female fetuses carrying these mutated paternal X chromosomes, the authors also suggest that these mutations could result in these X-bearing spermatozoa being unable to fertilize an ovum (Figure 2).

The only case in which ionizing radiation had a negative effect on the SSR value was observed in a retrospective study conducted in the Czech Republic from 1950 to 1999, which explicitly examined total live births and the SSR [113]. An analysis of monthly records found that the SSR only decreased in November 1986, seven months after the Chernobyl accident. Thus, a specific adverse impact of the Chernobyl disaster on male fetuses in the third month of prenatal growth was proposed. The researchers suggested a direct effect of radionuclides, such as iodine-131, on the loss of male fetuses. The concentration of iodine-131 in Czech radioactive clouds was exceptionally high following the accident [114]. Exposure to elevated levels of iodine-131 might cause severe hypothyroidism, leading to an increased rate of spontaneous abortions [115,116].

### 4.2. Other Nuclear Power Plants and Incidents

The proximity to nuclear facilities may affect the SSR, even in the absence of a nuclear accident. A retrospective cohort study was conducted on fathers employed at the Sellafield nuclear installation in England [117]. This analysis displayed a significantly heightened SSR among the offspring of the Sellafield workers. As the sex ratio is known to decrease with increasing paternal age [118], the fact that Sellafield fathers were more likely to fall within the 20–29-year age range may partially explain the observed increase in the SSR. A more pronounced impact was noted in males exposed to external ionizing radiation doses exceeding 10 mSv in the 90 days before conception. While this observation may be due to chance, stemming from dosage misclassification and multiple statistical tests, the researchers mentioned that they cannot disregard the potential for a statistical correlation between the SSR and paternal radiation exposure exceeding 10 mSv in the 90 days prior to conception. The authors also highlight that exposure to radiation in the 90 days before conception coincides with the phase of spermatogenesis; the exact biological effects of radiation during this phase remained unclear. Furthermore, within 35 km radii of nuclear facilities in Germany and Switzerland, there were marked increases in the SSR ranging from 0.30% to 0.40% during their operational periods [40]. Based on the existing literature, the authors suggested a potential selective mutagenic effect on paternal X gametes as a credible explanation for the impact of irradiation on the SSR and the diminished viability of female fetuses.

The identified correlation between the minimum distances between municipalities and nuclear facilities and the SSR in Germany and Switzerland aligns with the results obtained in another corresponding study conducted in France. Specifically, the SSRs of populations living within 35 km of 28 nuclear facilities in France were examined from 1968 to 2011 [119]. It was found that regions close to nuclear power plants showed higher SSRs, with the highest effect observed at distances of approximately 10–30 km. Consistent with previous studies, the authors suggest that irradiation may induce lethal mutations in the germline, which could affect female conceptuses and contribute to their culling. Likewise, irradiation can lead to nondisjunction phenomena, where chromosomes fail to separate during meiosis [120,121,122]. This condition could increase the prevalence of XO fetuses, which have a lower likelihood of intrauterine survival [123]. In a more recent study conducted by the same researchers, the examination of the SSR was extended to include data from French and German populations until 2017 [124]. A notable increase in the SSR was observed in 2016 at a distance of 36 km from the Centre de l’Aube Nuclear Disposal Facility, despite no recorded differences in the operation of the nuclear power plant. Additionally, a significant SSR upshift was observed in Germany following an incident at the Philippsburg nuclear plant in 2001, reducing male and female births by 5.56% and 6.92%, respectively.

In conclusion, the majority of studies agree that exposure to irradiation increases the SSR, either following a significant nuclear accident like the Chernobyl incident or due to proximity to nuclear power plants (Figure 2). In most cases, it is proposed that the mechanism involves mutations in the germline, especially paternal X gametes, caused by radiation. Thyroid dysregulation resulting in the culling of fetuses has also been mentioned in some cases.

## 5. Future Directions and Research Prospects

Moving forward, with technological advancements and improved research methods, future research in this field should delve deeper into the specific mechanisms through which natural disasters and nuclear accidents affect the SSR. This effort may involve examining genetic, hormonal, and environmental factors contributing to SSR alterations. Longitudinal studies tracking populations before, during, and after such events can provide invaluable insights into the lasting repercussions for reproductive health. Establishing long-term monitoring systems in regions affected by natural disasters or nuclear accidents can provide valuable data on how the secondary sex ratio evolves over time. Continuous observation can help identify trends, patterns, and potential implications for future generations considering the SSR as a health indicator.

It is imperative to meticulously consider confounding factors in future studies. For instance, variables such as parents’ socioeconomic status, seasonal fluctuations in the SSR, dietary habits, parents’ occupations, and other relevant factors must be carefully examined to avoid inaccurate conclusions. As research in this area progresses, ethical considerations regarding the implications of manipulating sex ratios or intervening in natural processes must be carefully assessed. Discussions on the social, cultural, and ethical dimensions of altering sex ratios in response to environmental stressors are crucial for shaping responsible policies and practices. Understanding the impacts of environmental stressors on the SSR can inform public health interventions aimed at mitigating potential risks. Policies focused on supporting maternal health pre- and post-conception, reducing environmental hazards, and enhancing disaster preparedness could contribute to more balanced sex ratios in at-risk populations. Collaboration between interdisciplinary teams, combining expertise from biology, epidemiology, and environmental science, will be key in unraveling the complexities of this intriguing area of study.

## 6. Conclusions

Natural disasters and nuclear accidents have been identified as potential trendsetters for the SSR. Precisely, the research findings indicate that earthquakes generally lower the SSR, primarily through the in utero selection process, due to the impact of psychological distress. Although the effects of cyclones, hurricanes, and floods on the SSR remain poorly understood, volcanic eruptions have shown a negative correlation with the SSR. On the other hand, the prevailing consensus is that radiation exposure tends to increase the SSR, whether stemming from a major nuclear incident like in Chernobyl or from proximity to nuclear facilities. The suggested pathophysiological process often involves radiation-induced mutations in paternal X gametes. Additionally, instances of thyroid dysregulation leading to fetal loss have been reported in specific cases.

## Figures and Tables

**Table 1:** Effects of cyclones, hurricanes, floods, and volcanic eruptions on secondary sex ratio.

Natural Disaster	Country	Date	SSR ^a^	Time to Alteration ^b^	Time of Effect ^c^	Parental Effect ^c^	Mechanism	Ref.
Cyclone Marcia	Australia	Feb.–Mar. 2015	Unaltered	-	-	-	-	[84]
Cyclone Yasi	Australia	Jan.–Feb. 2011	Increase	7–9 months	Gestation	Maternal	Increased female in utero mortality	[84]
Hurricane Katrina	USA	Aug. 2005	Unaltered	-	-	-	-	[87]
Hurricane Katrina	USA	Aug. 2005	Increase	8–10 months	Conception	Paternal; maternal	Increased radiation;lethal mutations in X gametes	[89]
Hurricane Katrina	USA	Aug. 2005	Unaltered	-	-	-	-	[88]
Brisbane flood	Australia	July 1965	Decline	10 months; 11 months	Conception	Paternal	Increased vulnerability of Y gametes	[90]
Pakistan floods	Pakistan	July–Sept. 2010; Aug.–Sept. 2011	Unaltered	-	-	-	-	[91]
Eyjafjallajökull eruption	Iceland	Apr. 2010	Decline	5 months; 7 months	Gestation	Maternal	In utero exposure to maternal stress; selection in utero	[92]
Laki eruption	Iceland	June 1783– Jan. 1784	Decline	Unspecified; in 1784	Gestation	Maternal	In utero exposure to maternal stress; selection in utero	[93]

^a^ Variations in the SSR that did not reach the level of significance are characterized as “Unaltered”. ^b^ The time needed from the beginning of each catastrophic event until a notable alteration in the SSR was observed. ^c^ Effects based on the potential mechanism of SSR alterations. SSR: secondary sex ratio, USA: United States of America, Jan.: January, Feb.: February, Mar.: March, Apr.: April, Aug.: August, Sept.: September.

## Data Availability

Not applicable.
